# CFNet: LiDAR-Camera Registration Using Calibration Flow Network

**DOI:** 10.3390/s21238112

**Published:** 2021-12-04

**Authors:** Xudong Lv, Shuo Wang, Dong Ye

**Affiliations:** School of Instrumentation Science and Engineering, Harbin Institute of Technology, Harbin 150001, China; 15B901019@hit.edu.cn (X.L.); 15B901018@hit.edu.cn (S.W.)

**Keywords:** LiDAR-camera calibration, deep learning, calibration flow

## Abstract

As an essential procedure of data fusion, LiDAR-camera calibration is critical for autonomous vehicles and robot navigation. Most calibration methods require laborious manual work, complicated environmental settings, and specific calibration targets. The targetless methods are based on some complex optimization workflow, which is time-consuming and requires prior information. Convolutional neural networks (CNNs) can regress the six degrees of freedom (6-DOF) extrinsic parameters from raw LiDAR and image data. However, these CNN-based methods just learn the representations of the projected LiDAR and image and ignore the correspondences at different locations. The performances of these CNN-based methods are unsatisfactory and worse than those of non-CNN methods. In this paper, we propose a novel CNN-based LiDAR-camera extrinsic calibration algorithm named CFNet. We first decided that a correlation layer should be used to provide matching capabilities explicitly. Then, we innovatively defined calibration flow to illustrate the deviation of the initial projection from the ground truth. Instead of directly predicting the extrinsic parameters, we utilize CFNet to predict the calibration flow. The efficient Perspective-n-Point (EPnP) algorithm within the RANdom SAmple Consensus (RANSAC) scheme is applied to estimate the extrinsic parameters with 2D–3D correspondences constructed by the calibration flow. Due to its consideration of the geometric information, our proposed method performed better than the state-of-the-art CNN-based methods on the KITTI datasets. Furthermore, we also tested the flexibility of our approach on the KITTI360 datasets.

## 1. Introduction

Environmental perception is an essential part of autonomous driving and robot navigation. Robust perception of the surrounding environment relies on various onboard sensors being installed on the mobile platform. The fusion of data from different sensors can improve perception. Light detection and ranging sensors (LiDAR) can obtain the spatial measurement of a scene with a wide frequency range and high accuracy. The sparse point clouds collected by the LiDAR lead to a low resolution of data, especially in the vertical orientation. The point clouds also lack color and texture information. Camera sensors can acquire high-resolution color images but are sensitive to illumination changes and cannot directly obtain depth information without other sensors. It is a common solution to utilize both LiDARs and cameras in perception systems. In this way, after the fusion of the point clouds and RGB images, the mobile platform can perceive either the geometric information or the corresponding semantic information. The effective fusion benefits the 3D object detection [1,2,3,4] and semantic mapping tasks [5,6,7]. Thus, extrinsic calibration between LiDARs and cameras, as the precondition of data fusion, has been a crucial scientific problem. However, extrinsic calibration is still challenging due to the laborious manual work, complicated environmental settings, specific calibration targets, and computationally expensive optimization.

Most LiDAR-camera calibration methods rely on specific calibration targets, such as a checkerboard or self-made targets. The matching relationships between 2D pixels and 3D point clouds are obtained by manual selection or automatic feature extraction, which are then used to calculate the extrinsic parameters. These offline calibration methods require complex environmental settings or well-designed targets. Burdensome human participation is demanded in this process, such as moving the targets or corresponding match selection. The environment setups and the target design mostly depend on the empirical goals. Reduplicated calibration works are requisites for real-world applications. Sensors’ mounting positions cannot be kept consistent. Vibrations and thermal strain in operation will cause displacement variations, introducing unpredictable deviations [8,9,10,11]. The pre-calibrated extrinsic parameters cannot be used in the ensuing data fusion procedure and should be re-calibrated. Although these offline methods can achieve satisfactory accuracy, they are time-consuming and laborious.

Several works of targetless and online methods were proposed to tackle the drawbacks of offline methods. Online methods work without any specific environmental settings and utilize the extracted human-designed features for estimating the extrinsic parameters by optimization. However, some assumptions about the environment limit the flexibility and the generalization of the online methods and make them unable to work in the absence of hand-crafted features. Besides, some approaches rely on accurate initial extrinsic parameters or additional motion information.

Recently, deep learning has been widely applied to learn image representations in high-level computer vision tasks instead of utilizing hand-crafted features. The achievements in these tasks have shown the superiority of feature extraction. Thus, some efforts attempted to adopt deep learning to learn the 6-DoF rigid transformation between LiDAR and camera data. Deep learning-based extrinsic calibration methods [12,13,14,15] also demonstrate flexibility and adaptability in changing environments. Some methods [12,13] cannot meet the performance requirements of real-world applications. Some methods [14,15] regress the parameters directly, which introduces training difficulties. The gap between the performances of deep learning-based methods and the practical demands is being bridged.

**Figure 1:**
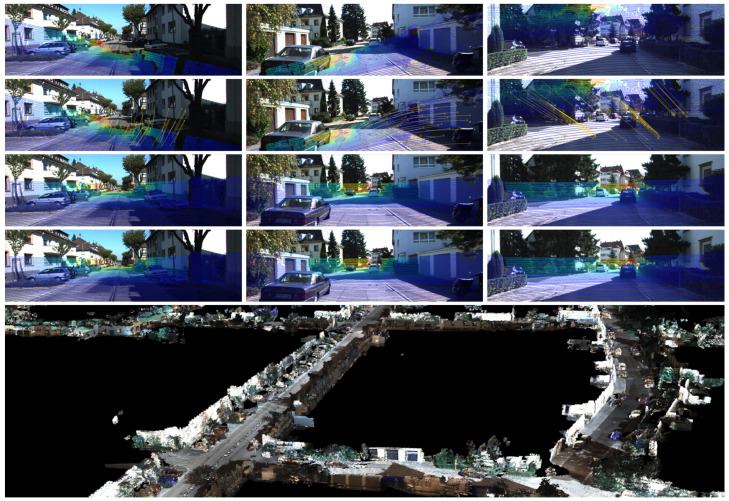
Examples of our proposed CFNet. (First Row) Mis-calibration. (Second Row) The predicted calibration flow. (Third Row) The calibration result predicted by CFNet. (Fourth Row) Ground truth. (Bottom Row) Three-dimensional colorized map generated by fusing the sensors using calibration values provided by our approach.

We first define a calibration flow, which represents the deviation between the positions of initial projected points and the ground truth, for automatic online LiDAR-camera self-calibration in this paper. The presented CFNet predicts a calibration flow instead of regressing extrinsic parameters. The predicted calibration flow is utilized to rectify the initial projected points for the construction of accurate 2D–3D correspondences. Then the EPnP algorithm [16] is used to calculate the final extrinsic parameters within the RANSAC scheme [17]. CFNet does not need additional specific calibration scenes, calibration targets, or initial calibration parameters, and is fully automatic. A similar idea was proposed in CMRNet++ [18], which is for monocular visual localization in LiDAR maps.An example of the utilization of CFNet is shown in Figure 1. More specifically, we showcase the contribution of CFNet as follows:

1. To the best of our Knowledge, CFNet is the first LiDAR-camera extrinsic calibration approach that introduces a geometric method to deep learning-based extrinsic calibration methods to predict 6-DoF transformation parameters. The results illustrate that CFNet is superior to many state-of-the-art deep learning-based calibration methods.
2. CFNet does not directly predict extrinsic parameters. We define a calibration flow as the output of CFNet which predicts the deviation between the positions of initial projected points and the ground truth. The calibration flow can shift the initial projection to the right positions and construct accurate 2D–3D correspondences.
3. To test the generalization of the proposed CFNet, we performed experiments on the KITTI360 benchmark datasets, which were collected by a different sensor system. After fine-tuning on a small dataset (4000 frames) with one epoch, the model could achieve a good performance. This test has not been used for previous deep learning-based methods.
4. The code will be publicly available at https://github.com/LvXudong-HIT/CFNet.

## 2. Related Works

According to whether they require specific calibration targets, the LiDAR-camera extrinsic calibration algorithms can be categorized into target-based and targetless. Target-based methods usually require calibration targets in an empty scene and estimate the extrinsic parameters by extracting the features from the targets. Targetless approaches extract hand-crafted feature from the environment to construct a penalty function for the calibration parameters. Some methods need additional information, such as initial values needed for optimization, or work under environmental assumptions. Deep learning-based calibration methods have been proposed in recent years. Taking advantage of the feature extraction capabilities of convolutional nerual networks (CNNs), learned representations can be employed to predict extrinsic parameters.

### 2.1. Target-Based Approach

The checkerboards are mainly used to calibrate cameras and are also suitable for the LiDAR-camera calibration [19,20,21,22,23]. The checkerboard’s pose in camera coordinates is calculated by triangulating the checkerboard’s corner points extracted from the image. In the LiDAR point clouds, the checkerboard is identified by the segmentation method, and the correlation between the plane points of LiDAR and the camera is established. With multiple checkerboards [24], an error function containing correlation constraints of multiple plane points can be established. To ensure the algorithm’s robustness, the checkerboards need to be evenly distributed in the sensor’s field of view (Angle and position). Verma et al. [25] extracted the central coordinates and the corresponding plane normal vector of the checkerboard as matching features. A genetic algorithm (GA) [26] was applied to acquire a globally optimal calibration result.

A systematic range of reflection bias is often observed in the LiDAR scanning results on the checkerboards, which leads to measurement errors and affects the final calibration results. Park et al. [27] used a monochromatic board as the calibration target instead of the checkerboard to deal with this issue. Dhall et al. [28] added the ArUco markers on the plate as the calibration target. Benefiting from the ArUco markers, the LiDAR-camera calibration task was transformed into a 3D-3D ICP problem. Guindel et al. [29] designed a custom-made calibration target that hollows out four circles on a rectangular board. The stereo camera and LiDAR detected the circles and fit the central coordinates of each of the four circles for the estimation of rigid body transformation. Beltrán et al. [30] pasted the ArUco markers on the calibration plate proposed in [29], which makes the new calibration target have the ability to calibrate LiDAR and monocular cameras. The LiDAR’s resolution is much lower than that of the camera, so the calibration methods based on the detection of the plane edge are restricted by the accuracy of LiDAR edge extraction.

Besides planar calibration targets, spherical targets are also appropriate for LiDAR-camera calibration [31,32,33]. Compared with planar targets, the advantage of the spherical target is that the camera can automatically detect the outline without depending on the camera’s angle of view and placement. Besides, sampling points of spherical objects can be detected conveniently on the LiDAR point cloud [33].

### 2.2. Targetless Approach

During the lifetimes of robots, the extrinsic parameters inevitably deviate to some extent. To ensure stable long-term operation, the robot needs to have the ability to detect a deviation and rectify the bias of each extrinsic parameter. Online calibration algorithms without specific calibration targets can effectively solve this problem.

When projecting the LiDAR point clouds to the image plane given correct extrinsic parameters, the 3D points with depth discontinuity will be more likely to be projected onto the edge of the image [34]. According with this hypothesis, Levinson et al. [35] proposed an online self-calibration method that contains an online deviation detection module and a rectification module. Another paper [36] introduced information theory into the calibration task. The rigid body transformation parameters are estimated by analyzing the statistical correlation between LiDAR and camera measurements. Mutual information (MI) was selected as the measurement metric of statistical correlation. Besides the 3D LiDAR point clouds and RGB images, the reflection intensity of LiDAR is also used to construct the MI objective function. Taylor et al. [37] leveraged a new measurement parameter, gradient orientation measure (GOM), to describe the gradient relation of LiDAR point clouds and images.

The above methods do not need calibration targets and are entirely data-driven calibration algorithms. Nevertheless, an appropriate initial calibration value is indispensable. A significant deviation between the initial value and the ground truth will bring about a wrong correlation. Therefore, these methods can only be used for fine-tuning calibration parameters. Ishikawa et al. [38] transformed the LiDAR-camera calibration into an extended problem of solving a hand-eye calibration problem. Hand-eye calibration can provide initial extrinsic parameters, but it depends heavily on visual odometry and LiDAR odometry accuracy [39]. A series of other studies [40,41,42] combined LiDAR-camera calibration with sensor fusion localization and mapping to establish a joint optimization function. The odometry and extrinsic parameters are optimized simultaneously for stable mapping.

### 2.3. Deep Learning Approach

It is inspiring to note that deep learning technology has made breakthroughs in many fields, such as classification, object detection, semantic segmentation, and object tracking. Some attempts have applied deep neural networks to multi-sensor calibration tasks. At present, the research in this field is still in the preliminary stage. To our knowledge, RegNet [13] was the first deep learning method that transformed feature extraction, feature matching, and global regression into real-time CNNs to deduce the six DOF of extrinsic parameters between LiDAR and camera data. RegNet ignores the geometric nature of se(3) using the quaternion distance as training loss. CalibNet [12] uses a 3D spatial transformer layer (3D STL) to deal with this problem. The output of the 3D STL is used to re-project the LiDAR point clouds to formulate a geometric loss. End-to-end training is performed by maximizing the geometric and photometric consistency between the input image and the point cloud. The above deep learning calibration methods ignore the tolerance within the error bounds. RGGNet [14] utilized the Riemannian geometry and deep generative model to build a tolerance-aware loss function.

Semantic information is introduced for obtaining an ideal initial extrinsic parameter. SOIC [43] exploits semantic information to calibrate and transform the initialization problem into the Perspective-n-Points (PnP) problem of the semantic centroid. Since the 3D semantic centroid of the point cloud and the 2D semantic centroid of the image cannot match accurately, a matching constraint cost function based on the semantic elements of the image and the LiDAR point cloud is also proposed. The optimal calibration parameter is obtained by minimizing the cost function. Zhu et al. [44] proposed an online calibration system that automatically calculates the optimal rigid-body motion transformation between two sensors by maximizing the mutual information of their perceived data without adjusting the environmental settings. By formulating the calibration as an optimization problem with semantic features, the temporally synchronized LiDAR and camera are registered in real-time.

## 3. Materials and Methods

**Figure 2:**
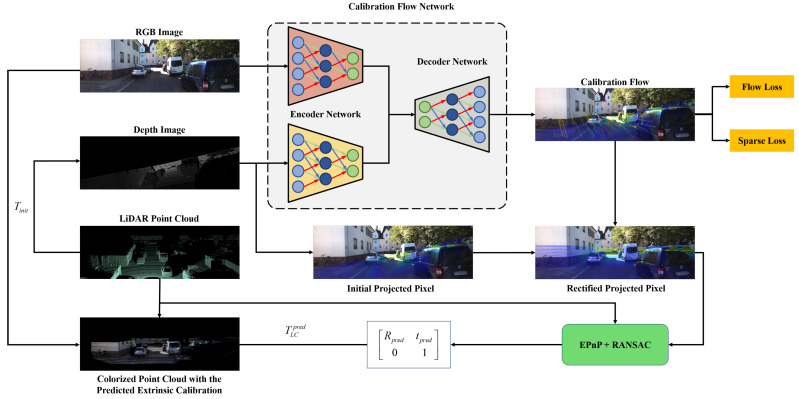
The overview of our proposed CFNet, an automatic online extrinsic calibration method that estimates the transformation parameters between 3D LiDAR and 2D camera. The calibration flow predicted by CFNet is utilized to correct the initial projected 2D coordinate in the image plane. After coordinate shift using calibration flow, the accurate 2D–3D correspondences between camera and LiDAR are detected. The extrinsic parameter is estimated by EPnP within the RANSAC scheme.

The workflow of our proposed CFNet is exhibited in Figure 2. In this section, we first describe the definition of the calibration flow. We then present our proposed calibration method based on calibration flow named CFNet, including the network architecture, loss functions, calibration inference, and the training details.

### 3.1. Calibration Flow

Given a group of LiDAR point clouds $L=\{P_{1},P_{2},\cdots ,P_{l}\}$, we can project each 3D point $P_{i}={\left[\begin{array}{ccc} X_{i} & Y_{i} & Z_{i} \end{array}\right]}^{T}\in R^{3}$ onto the image plane to obtain the corresponding 2D pixel coordinate $p_{i}={\left[\begin{array}{cc} u_{i} & v_{i} \end{array}\right]}^{T}\in R^{2}$ with LiDAR-camera extrinsic parameters $T_{gt}$ and the camera intrinsic matrix *K*. This projection process is expressed as follows:

(1) $$z_{i}\left[\begin{array}{c} u_{i}\\ v_{i}\\ 1 \end{array}\right]=K\left[R_{gt}|t_{gt}\right]\left[\begin{array}{c} X_{i}\\ Y_{i}\\ Z_{i}\\ 1 \end{array}\right]$$

(2) $$T_{gt}=\left[\begin{array}{cc} R_{gt} & t_{gt}\\ 0 & 1 \end{array}\right]$$

where $R_{gt}$ and $t_{gt}$ are the ground truth of the rotation matrix and the translation vector of the extrinsic calibration parameters $T_{gt}$. $z_{i}$ is the projected depth in the camera plane. After projection of LiDAR point clouds, not all points can be projected onto the image plane, so it is necessary to remove those invalid points according to the size of the image. For each projected 2D point $p_{i}$ that fulfills $0<u_{i}<W,0<v_{i}<H$ and the corresponding 3D LiDAR point $P_{i}$, we construct two valid point sets $q=\{p_{1},p_{2},\ldots ,p_{m}\}$ and $Q=\{P_{1},P_{2},\ldots ,P_{m}\}$, where *m* is the number of elements in the set.

If the given extrinsic parameters *T* have a deviation ΔT from the ground truth, that is, $T=\Delta T\cdot T_{gt}$, we call the points ${\tilde{p}}_{i}={\left[\begin{array}{cc} {\tilde{u}}_{i} & {\tilde{v}}_{i} \end{array}\right]}^{T}\in R^{2}$ projected with *T* the mis-calibrated projection points. Similarly, the verification of validity is also required for ${\tilde{p}}_{i}$ to construct valid point sets $\tilde{q}=\left\{{\tilde{p}}_{1},{\tilde{p}}_{2},\ldots ,{\tilde{p}}_{n}\right\}$ and $\tilde{Q}=\left\{P_{1},P_{2},\ldots ,P_{n}\right\}$, where *n* is the number of elements in the set. The mis-calibrated depth image *D* is acquired via z-buffer approach; each pixel ${\tilde{p}}_{i}$ in the image reserves the depth value $z_{i}$.

(3) $$D\left(\tilde{p_{i}}\right)=z_{i},\tilde{p_{i}}\in \tilde{q}$$

If the pixel in *D* does not have any matched LiDAR point, we set this pixel value to 0.

Due to the deviation ΔT between the extrinsic parameters *T* and the ground truth $T_{gt}$, the coordinate of the mis-calibrated projection point ${\tilde{p}}_{i}$ is different from $p_{i}$. We define the deviation between ${\tilde{p}}_{i}$ and $p_{i}$ as the calibration flow *f*. The calibration flow is similar to the optical flow, which includes two channels and represents the deviations of ${\tilde{p}}_{i}$ and $p_{i}$ in the horizontal and vertical directions, respectively. The ground truth calibration flow $f_{gt}$ is calculated as follows:

(4) $$f_{gt}\left(\tilde{p_{i}}\right)=\left[\begin{array}{cc} u_{i}-\tilde{u_{i}} & v_{i}-\tilde{v_{i}} \end{array}\right],\tilde{p_{i}}\in q_{f}$$

where $q_{f}$ is the valid point set of $f_{gt}$, which can be acquired by projecting the 3D points in the set $Q_{f}=Q⋂\tilde{Q}$; the invalid pixels are set to zero.

The concept of optical flow was first proposed in [45]. It is the instantaneous velocity of the pixel movement of the spatially moving object on the observation imaging plane. In an image sequence, optical flow uses the changes in the pixels and the correspondences between the previous frame and the current frame to calculate the motion of pixels in adjacent frames. Generally speaking, optical flow is caused by the movement of the foreground object itself, the movement of the camera, or the joint movement of the two in the scene. Similarly, we define the motion field of 2D projected point clouds on the image planes as the calibration flow. The assumption of calibration flow is that the intensity of point clouds is the same for both mis-calibrated and well-calibrated point clouds. Unlike optical flow, calibration flow is calculated at the same timestamp and is used to estimate the displacement of point clouds on the image plane during calibration.

### 3.2. The Architecture of CFNet

**Figure 3:**
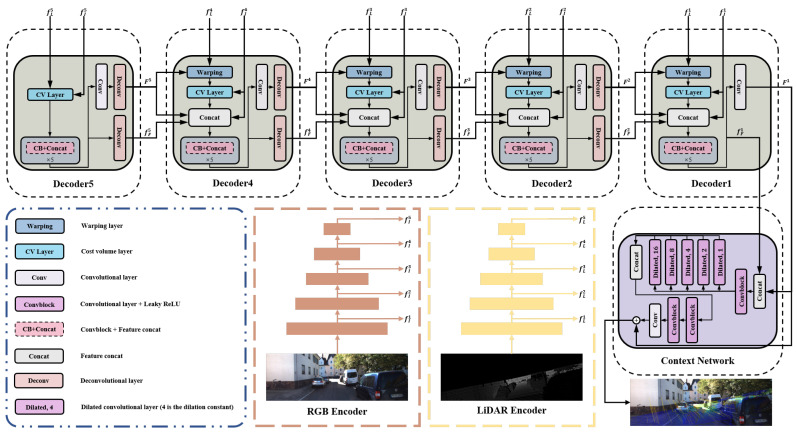
The architecture of our proposed calibration network CFNet.

As shown in Figure 3, CFNet is an encoder–decoder architecture. The encoder network includes two similar branches to extract multiscale features from the RGB image and the projected depth image. The multi-level decoder uses the multiscale features to predict the calibration flow progressively. We adopt a context network to generate a refined calibration flow at the end of the whole network. In this section, we introduce each component of CFNet.

#### 3.2.1. Encoder Network

There are two feature extraction networks based on a modified ResNet-18 [46] in the encoder: one is for RGB images (RGB encoder), and the other is for depth images projected from LiDAR point clouds (LiDAR encoder). We replaced the ReLU in both branches with Leaky ReLU. Since the inputs of the two branches are heterogeneous images, the weights are not shared between them. Since the projected depth image has only one channel, the number of input channels in the first convolution layer of the LiDAR encoder is 1. Besides that, the other parts of the feature extraction networks, such as the number of features per layer and stride, are consistent with the original ResNet-18. We do not apply the pre-trained models of ResNet-18 during training.

#### 3.2.2. Calibration Flow Decoder Network

The pyramid-like decoder, which has five levels, is utilized to generate calibration flow progressively. Each decoder consists of a feature warping layer, a cost volume layer, and a calibration flow estimator. The design of these parts is inspired by [47]. The five blocks named “Decoder” in Figure 3 are the calibration flow decoder network at different levels. We use the same network structure of the warping layer and the cost volume layer mentioned in [47]. The “CB-Concat” block in the calibration flow estimator is a five-layer convolutional network enhanced with DenseNet connections. The input and the output of the current convolutional layer construct the inputs of the next layer. The numbers of feature channels in each layer in the “CB-Concat” block are 128, 128, 96, 64, and 32. The outputs of each decoder **i** (i∈{1,…,5}) are the calibration flow $F^{i}$ and the corresponding flow features $f_{F}^{i}$.

It should be noted that the structures of Decoder 5 and Decoder 1 are different from the structures of the others. Decoder 5 does not have any calibration flow inputs. Thus, this level network computes the cost volume between the features of the RGB image and the depth image directly. Decoder 1 removes two deconvolutional layers from the network. The output of Decoder 1 is the input of the context network. In Table 1, we describe the parameters of each layer used in our calibration flow decoder network. In this table, **k** is the kernel size, **s** is the stride, **chns** is the number of output channels for each layer, **res** is the downscaling factor for each layer relative to the input image, and **input** is the input of each layer.

#### 3.2.3. Context Network

The context network is a multi-layer network for post-processing the calibration flow predicted by Decoder 1. This network is based on the dilated convolutions, which can effectively enlarge the receptive field size of each output unit at the desired pyramid level. It consists of five layers. The first and the third layer are convolutional layers with spatial kernels that are 1×1, whereas the kernel size of the last two convolutional layers is 3×3. The second layer of the context network consists of a group of dilated convolutional blocks with different dilation constants. From right to left, the dilation constants of these blocks are 1, 2, 4, 8, and 16. The outputs of these blocks are concatenated to new features as the input of the third layer. The production of the context network is a refined calibration flow *F*, which includes two channels. The parameters of each layer in the context network are described in Table 2. In this table, **d** is the dilated constant of the dilated convolutional layer, and the other notation is consistent with Table 1.

### 3.3. Loss Function

We use two loss functions during training: the supervised calibration flow loss $L_{cf}$ and the calibration flow sparse loss $L_{s}$.

#### 3.3.1. Supervised Calibration Flow Loss

After obtaining the predicted calibration flow, we inspect the sparse pixel-wise error between the predicted calibration flow *f* and the ground truth calibration flow $f_{gt}$ on valid pixels. We use the L1 norm for this supervised loss, and the error term is defined as

(5) $$L_{cf}=\frac{1}{N}\sum_{p}{∥f_{gt}\left(p\right)-f\left(p\right)∥}_{1},p\in q_{f}$$

where *N* is the number of elements in set $q_{f}$.

#### 3.3.2. Calibration Flow Sparse Loss

The sparse loss $L_{s}$ of the calibration flow is different from the optical flow. For dense optical flow, the correspondence flow maps are encouraged to be locally smooth, making the values of adjacent pixels close. Our proposed calibration flow is sparse, and most of the pixels are invalid. Thus, the function of the smoothness loss $L_{s}$ is to enforce the displacements of pixels without ground truth to be similar to the ones of the neighboring pixels.

(6) $$L_{s}=\frac{1}{M}\sum_{p}S\left(u,v\right),p\in {\hat{q}}_{f}$$

(7) $$\begin{array}{cc} S(u,v) & =\rho (f(u,v)-f(u+1,v))\\ \; & +\rho (f(u,v)-f(u,v+1)) \end{array}$$

where ${\hat{q}}_{f}$ is the invalid point set of $f_{gt}$, *M* is number of elements in the set ${\hat{q}}_{f}$, and ρ is the generalized Charbonnier function $\rho \left(x\right)={\left(x^{2}+\varepsilon^{2}\right)}^{\alpha },\varepsilon =10^{-9},\alpha =0.25$, as in [48].

Our final loss function consists of a weighted sum of the supervised calibration flow loss and calibration flow sparse loss:

(8) $$L_{total}=\lambda L_{cf}+\left(1-\lambda \right)L_{s}$$

where λ=0.9.

### 3.4. Calibration Inference

We make shift a correction for the initial 2D projection point ${\tilde{p}}_{i}$ using calibration flow. The shift correction process is as follows:

(9) $${p_{i}}^{\prime }={\tilde{p}}_{i}+f\left({\tilde{p}}_{i}\right),{\tilde{p}}_{i}\in \tilde{q}$$

where ${p_{i}}^{\prime }={\left[\begin{array}{cc} {u_{i}}^{\prime } & {v_{i}}^{\prime } \end{array}\right]}^{T}\in R^{2}$ is the rectified 2D coordinate of the valid projected point ${\tilde{p}}_{i}$. What should be noted is that the rectified point $p_{i}^{\prime }$ may lie outside the image plane. Therefore, the validity of $p_{i}^{\prime }$ also needs to be checked to fulfill $0<{u_{i}}^{\prime }<W,\;0<{v_{i}}^{\prime }<H$. After acquiring a set of valid 2D–3D correspondences, we transfer the LiDAR-camera extrinsic calibration task to a Perspective-n-Point (PnP) problem. We use the EPnP [16] within the RANSAC scheme to solve this problem, with a maximum of 10 iterations, 5 repeats, and an inlier threshold value of 1 pixel.

**Figure 4:**
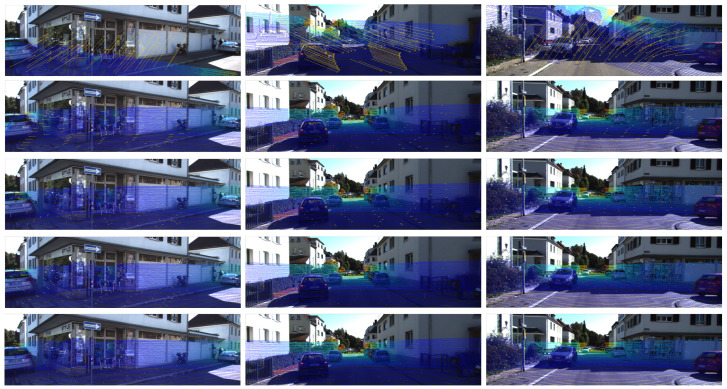
Examples of the predicted calibration flow represented by yellow arrows. Each row of pictures shows the calibration flows predicted by the models of different initial error ranges, from $N_{1}$ to $N_{5}$ (from top to bottom).

Similarly to LCCNet [15], we employ a multi-range iterative refinement method to improve the calibration accuracy further. We train five models with different initial error ranges, $N_{1}∼(\pm$1.5 m, $\pm 20{}^{\circ })$, $N_{2}∼(\pm$1.0 m, $\pm 10{}^{\circ })$, $N_{3}∼(\pm$0.5 m, $\pm 5{}^{\circ })$, $N_{4}∼(\pm$0.2 m, $\pm 2{}^{\circ })$, and $N_{5}∼(\pm$0.1 m, $\pm 1{}^{\circ })$ respectively. The calibration iterative refinement process is shown in Algorithm 1. The inputs are camera frame *I*, LiDAR point clouds *L*, camera intrinsic *K*, and the initial calibration parameters *T*. After pre-processing the sensor data, we project the LiDAR point clouds *L* onto the image plane to generate the sparse depth image *D* and the valid projected 2D points set $\tilde{q}$. Due to the input size of the network being 320×960, we need to crop the original RGB image and the depth image simultaneously. To ensure the cropped depth image $D^{\prime }$ contains as many points as possible, we calculate the centroid of $\tilde{q}$ to get the location of the crop window. Then, we use the output of $N_{1}∼(\pm$1.5 m, $\pm 20{}^{\circ })$ to rectify the coordinate of each projected point ${\tilde{p}}_{i}$. The rectified 2D projected points and the corresponding valid LiDAR point clouds construct new 2D–3D correspondences. By applying the EPnP algorithm within the RANSAC scheme, we calculate the extrinsic parameters $T_{pred}$ and set them to $T_{1}$. We regard the transformation $T_{1}$ as a new initial extrinsic parameters to re-project the LiDAR point clouds and generate a depth image, which is taken as the input of $N_{2}∼(\pm$1.0 m, $\pm 10{}^{\circ })$. The number of accurate 2D–3D correspondences in the current iteration is much more than the last iteration in theory. The above process is repeated five times, iteratively improving the extrinsic calibration accuracy. The calibration flows predicted by networks with different initial error ranges are illustrated in Figure 4. The inference time for a single iteration on one NVIDIA TITAN RTX GPU is about 40 ms for the calibration flow prediction and 35 ms for EPnP+RANSAC.

**Algorithm 1:** Calibration iterative refinement

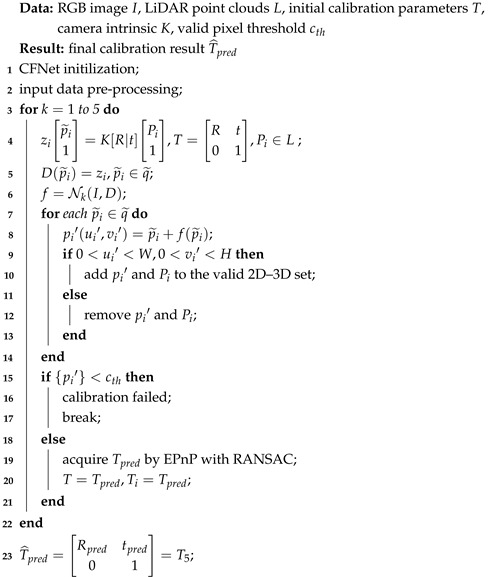



The method we propose above only uses one pair of RGB image LiDAR point cloud to predict the extrinsic calibration parameters. In practical applications, inaccurate calibration flow will result in false calibration parameters. If we analyze the results over a sequence using the median value as the reference, the abnormal values that are significantly different from the reference can be eliminated.

## 4. Experiments and Results

### 4.1. Dataset Preparation

We evaluated our approach on the KITTI benchmark datasets [49], which include RGB images and Velodyne point cloud recordings collected from different scenes. The timestamps of the LiDAR and camera are synchronized, so the images and point clouds in each sequence are paired. We define the extrinsic parameters’ ground truth $T_{gt}$ between LiDAR and camera as the transformation matrix from the camera coordinate to the LiDAR coordinate. By adding a random variable ΔT, we can obtain the initial calibration parameters $T=\Delta T\cdot T_{gt}$. The LiDAR point clouds are projected onto the image plane with *T* and the camera intrinsic matrix *K* to generate the mis-calibrated depth image *D*. The network takes an RGB image *I* and the corresponding depth image *D* as input. The calibration flow ground truth can be obtained by Equation (4).

We used the raw recordings from the KITTI dataset, specifically the left color image and Velodyne point clouds recordings. We used all drives (except 0005 and 0070 drive) in sequence 2011_09_26 for training and validation. The initial calibration off-range ΔT was (±1.5 m, $\pm 20{}^{\circ })$. To compare our method with other learning-based (CNN-based) methods, we utilized the same four test datasets [14] on the raw recordings of the KITTI dataset. Each test dataset was independent of the training dataset with the following test name configurations:

**T1:** 0028 drive in 2009_09_30 sequence, with initial off-range as (±1.5 m, $\pm 20{}^{\circ })$ and (±0.2 m, $\pm 20{}^{\circ })$.

**T2:** 0005/0070 drive in 2009_09_26 sequence, with initial off-range as (±0.2 m, $\pm 15{}^{\circ })$.

**T3:** 0005/0070 drive in 2009_09_26 sequence, with initial off-range as (±0.2 m, $\pm 10{}^{\circ })$.

**T4:** 0027 drive in 2009_10_03 sequence, with initial off-range as (±0.32 m, $\pm 2{}^{\circ })$.

Due to the image dimensiosn in the KITTI benchmark dataset being different (ranging from 1224×370 to 1242×376), pre-processing was required to resize them to a consistent size. To fulfill the input size of the network, so that the input width and height were multiples of 32, we randomly cropped the original image to 960×320. We generated the sparse depth image by projecting the LiDAR point cloud onto the original image plane and then cropped the original RGB image and the sparse depth image simultaneously. Then the inputs of CFNet could be obtained without changing the camera data’s intrinsic parameters. Data augmentation was performed on the cropped input data. We added color augmentations with a 50 chance, where we performed random brightness, contrast, saturation, and hue shifts by sampling from uniform distributions in the ranges of [0.7,1.3] for brightness, contrast, and saturation, $\left[1-0.3/3.14,1+0.3/3.14\right]$ for hue shifts.

KITTI360 datasets [50] were utilized to test our proposed LiDAR-camera calibration algorithm CFNet as well. We fine-tuned the models trained on the raw KITTI recordings with 4000 frames from drive 0000 in 2013_05_28 sequence of the KITTI360 datasets. Other sequences were selected as test datasets.

### 4.2. Training Details

During the training, we used Adam [51] with an initial learning rate of 1 ×$10^{-3}$. We set the parameters of the Adam solver to the default values $\beta_{1}=0.9$, $\beta_{2}=0.999$ and $\epsilon =10^{-8}$. We trained the CFNet on four NVIDIA TITAN RTX GPUs with batch size 100 and total epochs 80. For the multi-range network, it is not necessary to retrain each network from scratch. Instead, a large-range model can be regarded as the pre-trained model for small-range model training to speed up the training process. The number of training epochs of the model with the largest range was set to 80, and the others were set to 50. The network weights were initialized using kaiming initialization [52]. We fine-tuned the pre-trained model on KITTI360 datasets with 1 epoch or 10 epochs to test the generalization of CFNet.

### 4.3. Evaluation Metrics

We analyzed the calibration results according to the rotation and the translation errors of the predicted extrinsic parameters. The translation error was evaluated by the Euclidian distance between the translation vectors. The absolute translation error is expressed as follows:

(10) $$E_{t}=\left|t_{pred}-t_{gt}\right|$$

We tested the absolute translation error in X,Y,Z directions, $E_{X},E_{Y},E_{Z}$; and the mean value $\bar{t}=\left(E_{X}+E_{Y}+E_{Z}\right)/3$. To test the angle error of the extrinsic rotation matrix on three rotation axis, we need to transform the rotation matrix to Euler angles and compute the angle error according roll $E_{Roll}$, pitch $E_{Pitch}$, and yaw $E_{Yaw}$, and the mean value $\bar{R}=\left(E_{Roll}+E_{Pitch}+E_{Yaw}\right)/3$.

(11) $$R_{pred}^{-1}\cdot R_{gt}=\left[\begin{array}{ccc} r_{11} & r_{12} & r_{13}\\ r_{21} & r_{22} & r_{23}\\ r_{31} & r_{32} & r_{33} \end{array}\right]$$

(12) $$\begin{array}{cc} \; & E_{Yaw}=\theta_{z}=atan2\left(r_{21},r_{11}\right)\\ \; & E_{Pitch}=\theta_{y}=atan2\left(-r_{31},\sqrt{r_{31}^{2}+r_{33}^{2}}\right)\\ \; & E_{Roll}=\theta_{x}=atan2\left(r_{32},r_{33}\right) \end{array}$$

Besides, we applied another two se(3) error based evaluation metrics mentioned in [14]: mean error (MSEE) se(3) and mean re-calibration rate (MRR). MRR reflects how much noise us compensated by the re-calibration.

### 4.4. Results and Discussion

#### 4.4.1. The Calibration Results with Random Initialization

The calibration results on the raw KITTI recordings are shown in Table 3 and Table 4. To compare the performance of CFNet with those of RegNet and CalibNet, we set two test datasets with different initial off-range as (±1.5 m, $\pm 20{}^{\circ })$ and (±0.2 m, $\pm 20{}^{\circ })$ according to the experimental settings of RegNet and CalibNet. Both of these two calibration methods adopt a similar iterative refinement algorithm to that described in Section 3.4. As shown in Table 3, CFNet achieved a mean translation error of 0.995 cm (X, Y, Z: 1.025, 0.919, 1.042 cm) and a mean angle error of $0.087{}^{\circ }$ (roll, pitch, yaw: $0.059{}^{\circ }$, $\;0.110{}^{\circ }$, $\;0.092{}^{\circ }$) at initial off-range (±1.5 m, $\pm 20{}^{\circ })$. It is obvious that CFNet is far superior to RegNet and CalibNet.

**Figure 5:**
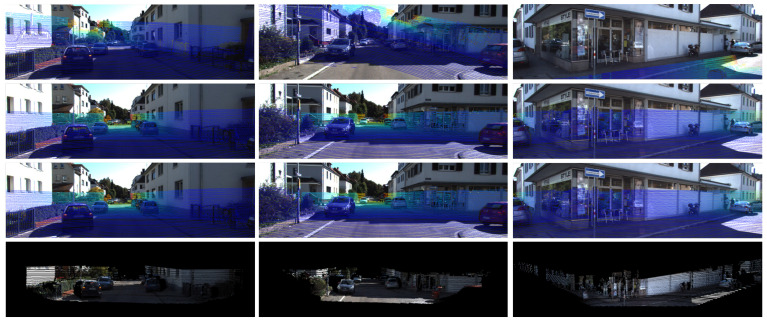
Examples of the calibration results on the T1 test dataset. The first row shows the mis-calibrated LiDAR point clouds projected onto the image plane given initial extrinsic parameters. The second row shows the projected LiDAR point clouds using CFNet’s prediction, and the third row shows the corresponding ground truth. The last row shows the colorized point cloud with the predicted extrinsic parameters.

Figure 5 shows some examples of the CFNet predictions. It can be seen that the projected depth image generated by the predicted extrinsic calibration parameters is basically the same as the ground truth. The last row’s colorized point cloud also shows that the projected LiDAR point clouds align accurately with the RGB image. Our proposed CFNet can predict the LiDAR-camera extrinsic parameters accurately at different initial values and in different working scenes.

The comparison results from the test datasets T2, T3, and T4 shown in Table 4 also illustrate the superiority of CFNet. Compared to the translation errors and the rotation errors utilized as the metrics in the above experiments, se3 error [14] is a more direct evaluation metric. The initial off-range result of the test dataset T3 is smaller than that of the test dataset T2. The MSEE for β-RegNet and RGGNet are smaller for T3 than T2. Nevertheless, the MSEE are the same for CFNet using those two test datasets, proving that CFNet is more robust than β-RegNet and RGGNet with different off-range settings. For test dataset T4, the performance of β-RegNet degraded heavily, and RGGNet needed to re-train on an additional dataset by adding a small number of data from 2009_10_03 sequence to achieve a good calibration result, $0.010\left(83.22\%\right)$. CFNet did not need any additional training dataset and re-training process. The calibration results $0.001\left(98.08\%\right)$ demonstrate that CFNet generalizes well. Thus, CFNet outperforms most of the state-of-the-art learning-based calibration algorithms and even the motion-based calibration method. We can also see that compared to the motion-based algorithm [39], our proposed method performs better, without the requirements of hand-crafted features or the extra IMU sensor.

#### 4.4.2. Generalization Experiments

**Figure 6:**
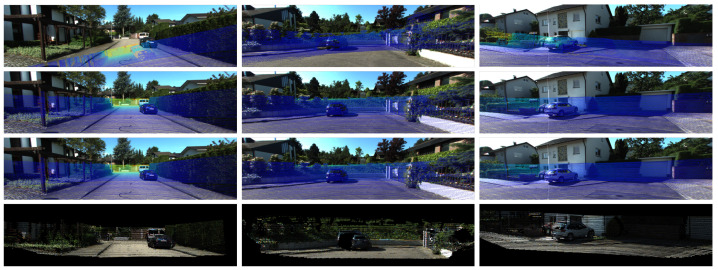
Examples of the calibration results on the KITTI360 recordings. (First Row) Initial calibration. (Second Row) Calibration result. (Third Row) Ground truth. (Forth Row) Colorized point cloud with the predicted extrinsic parameters.

We also tested the performance of CFNet on the KITTI360 benchmark dataset. Due to the sensors’ parameters having changed, CFNet needed to be re-trained to fit the new sensor settings. We utilized 4000 frames from drive 0000 in the 2013_05_28 sequence of the KITTI360 datasets to fine-tune the pre-trained models trained on the raw KITTI dataset. The evaluation results on the KITTI360 datasets are shown in Table 5 and Figure 6. Despite re-training on a tiny sub dataset with ten epochs, excellent results were obtained on the test sequences. Therefore, an excellent prediction model can be obtained with fast re-training when the sensor parameters change, such as the camera focal length and the LiDAR-camera extrinsic parameters. From Table 5, it is easy to see that more training epochs will obtain better calibration results. However, the results obtained after training one epoch are also acceptable.

#### 4.4.3. The Calibration Results in a Practical Application

In practical applications, the initial extrinsic parameters will not vary as widely as in the previous experiments. Thus, in this part, we validated the performance of the CFNet in practice. The initial extrinsic parameters of a well-designed sensor system are fixed values. The initial extrinsic parameters can be obtained by measurement or estimation. In this part, we took the assembly positions of each sensor in the sensor system as the initial values. The KITTI datasets and the KITTI360 datasets both provide these values, which can be used directly. In addition, estimating extrinsic parameters by a single frame will lead to outliers. Thus, we utilized multiple-image LiDAR sequences to predict extrinsic parameters of each frame. The median filtering algorithm was implemented for deleting the outliers, and the median value was regarded as the final extrinsic calibration estimation. By changing the length of sequences, we also tested the impacts of the length of sequence on estimating the extrinsic parameters and how to choose the appropriate length of sequences in practice. In the experiments, we randomly selected 20 sequences of fixed length from the test datasets, and took the assembly positions of sensors as the initial values for CFNet predicting extrinsic calibration parameters.

**KITTI Odometry datasets.** Each sequence in the KITTI Odometry datasets has different extrinsic calibration parameters. Therefore, we evaluated our CFNet on the KITTI Odometry datasest to test whether CFNet works with different extrinsic calibration parameters in various scenes. We selected sequences 00, 01, 08, 12, and 14 from the KITTI Odometry datasets as test data. Sequences 00 and 08 were collected in an urban area, so they include lots of buildings, pedestrians, and vehicles. Sequences 01 and 12 captured images of a highway with many vehicles moving at high speeds. For sequence 14, the data were collected in the city’s suburbs, thereby containing a large amount of vegetation and no buildings and vehicles.

**Figure 7:**
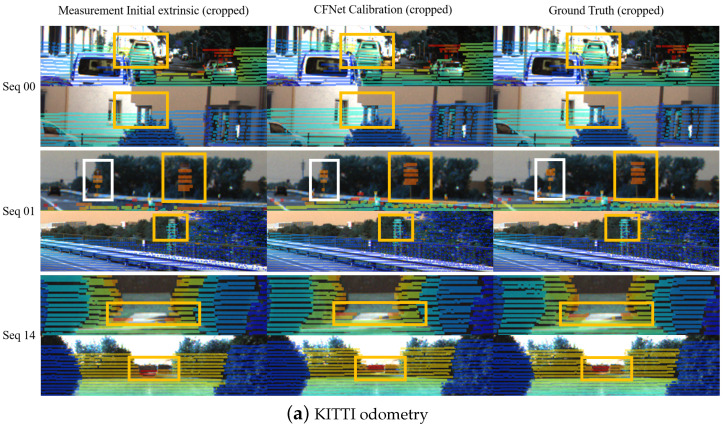

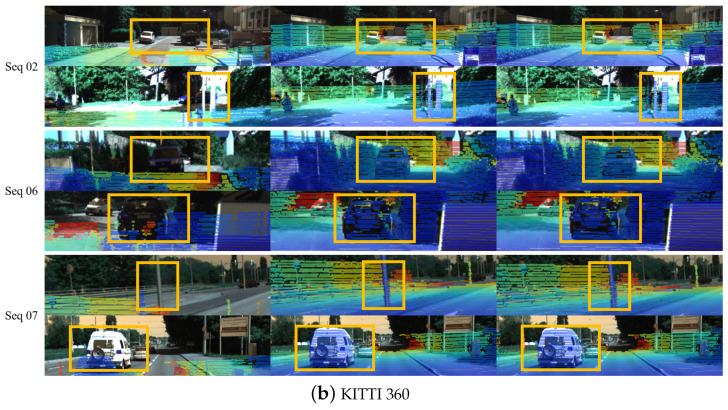
Examples of the calibration results with measurement initial extrinsic parameters using the KITTI odometry and KITTI 360 datasets. Each row represents a different test sequence. Images were cropped for better visualization, and the reference objects are shown in the rectangles.

**Figure 8:**
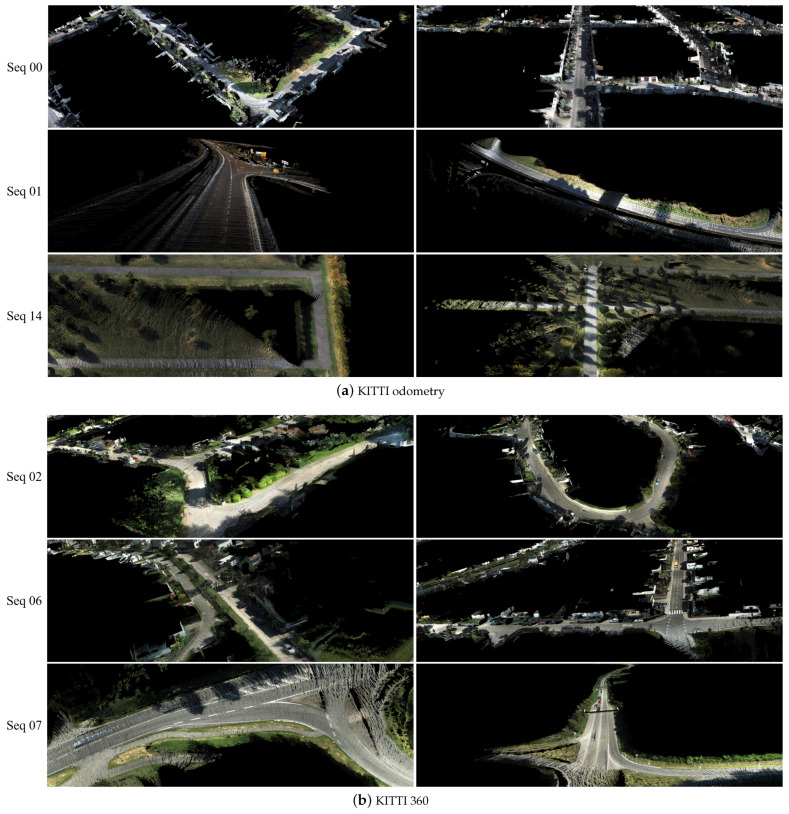
Examples of the reconstructed 3D colorized maps created by fusing the data of LiDAR and camera from the KITTI odometry and KITTI 360 datasets. The extrinsic parameters were provided by CFNet, with the initial parameters from measurements.

The calibration results in Table 6 illustrate that the CFNet can obtain accurate calibration results given initial parameters. The calibration errors for sequences 00 and 08 are a bit lower than those of the other three sequences. This is because most of the scenes in the training dataset belonged to the urban category, leading to a slight performance reduction in different scenes. By changing the length of sequences, we acquired a group of calibration results for analysis. In most cases, the mean translation error decreased when the length increased, and the mean rotation error had a small range of fluctuation. Although the sequence length influences the calibration results, the differences between different length settings are negligible. Therefore, a short sequence length, e.g., 10, is enough for a practical application. Figure 7a shows visualized results from the predicted extrinsic calibration parameters in different scenes. Due to the initial values from assembly positions of sensors being close to the ground truth, the initial calibration error is small. However, CFNet still estimated the best extrinsic parameters and found correct correspondences between RGB pixels and LiDAR points. With the extrinsic calibration parameters estimated by the proposed CFNet, we constructed 3D colorized maps by fusing RGB images and exhibit them in Figure 8a.

**KITTI360 datasets.** We also implement the proposed CFNet on KITTI360 datasets. All the sequences in the KITTI360 datasets have the same extrinsic calibration parameters. As shown in Table 7, the deviation in the initial extrinsic parameters is large in the rotation part ($2.73^{\circ }$). Sequences 0002 and 0006 were captured in urban areas, so they include constructions, pedestrians, and vehicles. Sequence 0007, which only has high-speed moving vehicles and trees, was collected by a highway. It can be noticed that, after training a few epochs with new data, CFNet could accurately estimate extrinsic parameters for a new sensor system. Similarly to the KITTI odometry datasets, the deviations between different sequence lengths are tiny. In Figure 7b, we exhibit some visualized examples of the calibration results predicted by CFNet. The 3D colorized fusion maps generated by predicted extrinsic calibration parameters from CFNet are shown in Figure 8b.

## 5. Conclusions

In this paper, we proposed a novel supervised calibration approach called CFNet for the estimation of a 6-DoF extrinsic transformation between a 3D LiDAR and a 2D camera. To improve the accuracy and the generalization of the CNN-based LiDAR-camera calibration methods that regress the extrinsic parameters directly, CFNet predicts the calibration flow to rectify the projected coordinates of the mis-calibrated LiDAR point clouds. Inspired by the optical flow, the calibration flow is presented to describe the deviations between the initial mis-calibrated projection and the ground truth. After rectification, a group of accurate 2D–3D correspondences are constructed and the extrinsic matrix is calculated by EPnP algorithm with the RANSAC scheme.

The experiments demonstrated that CFNet is superior to many state-of-the-art CNN-based and optimization-based methods. In practical applications, CFNet can be easily migrated to new scenarios and new sensor systems. With a few training epochs on the new sensor data, one can obtain an ideal fine-tuned model. In addition, the experimental analysis of the length of the calibration sequence showed that the length of the sequence has little influence on the final result. Therefore, the best calibration result can be obtained with a short operation time in a practical application.

## Figures and Tables

**Table 1:** Network architecture of the calibration flow decoder network of our proposed CFNet.

Calibration Flow Decoder							
Layer		k	s	chns	res	Input	Activation
Decoder5	CV Layer5	-	-	81	1/32	$f_{I}^{5},f_{L}^{5}$	Leaky ReLU
	CB-Concat5	3	1	529	1/32	CV Layer5	Leaky ReLU
	Conv5	3	1	2	1/32	CB-Concat5	-
	Deconv5_1	4	2	2	1/16	Conv5	-
	Deconv5_2	4	2	2	1/16	CB-Concat5	-
Decoder4	Warping4	-	-	256	1/16	$F^{5},f_{L}^{4}$	-
	CV Layer4	-	-	81	1/16	$f_{I}^{4}$, Warping4	Leaky ReLU
	CB-Concat4	3	1	789	1/16	CV Layer4, $f_{I}^{4},F^{5},f_{F}^{5}$	Leaky ReLU
	Conv4	3	1	2	1/16	CB-Concat4	-
	Deconv4_1	4	2	2	1/8	Conv4	-
	Deconv4_2	4	2	2	1/8	CB-Concat4	-
Decoder3	Warping3	-	-	128	1/8	$F^{4},f_{L}^{3}$	-
	CV Layer3	-	-	81	1/8	$f_{I}^{3}$, Warping3	Leaky ReLU
	CB-Concat3	3	1	661	1/8	CV Layer3, $f_{I}^{3},F^{4},f_{F}^{4}$	Leaky ReLU
	Conv3	3	1	2	1/8	CB-Concat3	-
	Deconv3_1	4	2	2	1/4	Conv3	-
	Deconv3_2	4	2	2	1/4	CB-Concat3	-
Decoder2	Warping2	-	-	64	1/4	$F^{3},f_{L}^{2}$	-
	CV Layer2	-	-	81	1/4	$f_{I}^{2}$, Warping2	Leaky ReLU
	CB-Concat2	3	1	597	1/4	CV Layer2, $f_{I}^{2},F^{3},f_{F}^{3}$	Leaky ReLU
	Conv2	3	1	2	1/4	CB-Concat2	-
	Deconv2_1	4	2	2	1/2	Conv2	-
	Deconv2_2	4	2	2	1/2	CB-Concat2	-
Decoder1	Warping1	-	-	64	1/2	$F^{2},f_{L}^{1}$	-
	CV Layer1	-	-	81	1/2	$f_{I}^{1}$, Warping1	Leaky ReLU
	CB-Concat1	3	1	597	1/2	CV Layer1, $f_{I}^{1},F^{2},f_{F}^{2}$	Leaky ReLU
	Conv1	3	1	2	1/2	CB-Concat1	-

**Table 2:** Network architecture of the context network of our proposed CFNet.

Context Network						
Layer	k	s, d	chns	res	Input	Activation
Convblock1	1	1, 1	128	1/2	$F^{1},f_{F}^{1}$	Leaky ReLU
Dilated1	3	1, 1	64	1/2	Convblock1	Leaky ReLU
Dilated2	3	1, 2	64	1/2	Convblock1	Leaky ReLU
Dilated3	3	1, 4	64	1/2	Convblock1	Leaky ReLU
Dilated4	3	1, 8	64	1/2	Convblock1	Leaky ReLU
Dilated5	3	1, 16	64	1/2	Convblock1	Leaky ReLU
Convblock2	1	1, 1	64	1/2	Dilated1∼5	Leaky ReLU
Convblock3	3	1, 1	32	1/2	Convblock2	Leaky ReLU
Conv1	3	1, 1	2	1/2	Convblock3	-

**Table 3:** The comparison results on the T1 test dataset.

Methods	Mis-Calibration Range	Translation (cm)				Rotation (${}^{\circ }$)			
		$E_{X}$	$E_{Y}$	$E_{Z}$	$\bar{t}$	$E_{\mathrm{Roll}}$	$E_{\mathrm{Pitch}}$	$E_{\mathrm{Yaw}}$	$\bar{R}$
RegNet	(±1.5 m, $\pm 20{}^{\circ })$	7	7	4	6	0.24	0.25	0.36	0.28
Calibnet	(±0.2 m, $\pm 20{}^{\circ })$	4.2	1.6	7.22	4.34	0.15	0.9	0.18	0.41
CFNet	(±1.5 m, $\pm 20{}^{\circ })$	**1.025**	**0.919**	**1.042**	**0.995**	**0.059**	**0.110**	**0.092**	**0.087**
	(±0.2 m, $\pm 20{}^{\circ })$	**0.463**	**1.230**	**0.802**	**0.831**	**0.028**	**0.125**	**0.105**	**0.086**

**Table 4:** The comparison results using the T2, T3, and T4 test datasets.

Method	T2 (MSEE/MRR)	T3 (MSEE/MRR)	T4 (MSEE/MRR)
TAYLOR [39]	*	*	0.010(†)
CalibNet [12]	-	0.022/-	*
β-RegNet [14]	0.048/53.23%	0.046/34.14%	0.092/−1.89%
RGGNet [14]	0.021/78.40%	0.017/72.64%	0.010/83.22%
CFNet	**0.003/96.74%**	**0.003/94.53%**	**0.001/98.08%**

* means the author does not provide the result in his paper. - shows that the calibration algorithm fails. † represents that the calibration algorithm does not have this metric.

**Table 5:** The comparison of the calibration errors on KITTI360 datasets with different training epochs.

Sequence	Training 1 Epoch						Training 10 Epoch					
	Translation (cm)			Rotation (${}^{\circ }$)			Translation (cm)			Rotation (${}^{\circ }$)		
	$E_{X}$	$E_{Y}$	$E_{Z}$	$E_{\mathrm{Roll}}$	$E_{\mathrm{Pitch}}$	$E_{\mathrm{Yaw}}$	$E_{X}$	$E_{Y}$	$E_{Z}$	$E_{\mathrm{Roll}}$	$E_{\mathrm{Pitch}}$	$E_{\mathrm{Yaw}}$
0002	1.130	3.390	2.724	0.247	0.211	0.128	**0.834**	**1.270**	**0.619**	**0.099**	**0.048**	**0.047**
0003	3.076	1.538	3.534	0.117	0.044	0.046	**0.872**	**1.078**	**0.800**	**0.042**	**0.038**	**0.046**
0004	2.751	4.68	2.822	0.168	0.340	0.081	**1.026**	**2.165**	**0.823**	**0.145**	**0.218**	**0.050**
0005	3.840	0.967	4.595	0.314	0.364	0.479	**1.286**	**1.830**	**1.073**	**0.111**	**0.190**	**0.246**
0006	2.917	2.646	5.090	0.195	0.238	0.281	**0.435**	**0.583**	**1.275**	**0.147**	**0.017**	**0.122**
0007	3.325	2.003	3.202	0.280	0.244	0.314	**0.808**	**1.410**	**1.175**	**0.040**	**0.153**	**0.116**
0009	1.958	3.379	2.565	0.124	0.088	0.085	**0.514**	**1.257**	**1.303**	**0.062**	**0.078**	**0.054**
0010	1.460	3.262	1.657	0.072	0.133	0.235	**1.214**	**1.565**	**1.533**	**0.071**	**0.088**	**0.077**

**Table 6:** The calibration results given initial extrinsic parameters using the KITTI odometry dataset.

Sequence	Test Length	Translation (cm)				Rotation (${}^{\circ }$)			
		$E_{X}$	$E_{Y}$	$E_{Z}$	$\bar{t}$	$E_{\mathrm{Roll}}$	$E_{\mathrm{Pitch}}$	$E_{\mathrm{Yaw}}$	$\bar{R}$
00	10	0.254	0.209	0.457	0.306	**0.082**	0.035	**0.044**	0.053
	20	**0.185**	**0.184**	0.314	**0.227**	**0.082**	**0.030**	**0.044**	**0.052**
	50	0.212	0.222	**0.311**	0.248	0.097	0.038	0.050	0.061
	100	0.233	0.235	0.329	0.265	0.112	0.041	0.056	0.069
	Initial Extrinsic Parameters	0.809	2.449	2.160	1.806	0.413	0.025	0.463	0.300
01	10	**0.396**	0.155	1.301	0.617	0.080	0.219	**0.061**	0.12
	20	0.512	**0.088**	1.175	0.591	**0.076**	**0.207**	0.069	**0.117**
	50	0.525	0.091	1.058	0.558	0.085	0.211	0.080	0.125
	100	0.508	0.110	**0.973**	**0.530**	0.093	0.219	0.094	0.135
	Initial Extrinsic Parameters	0.809	2.449	2.160	1.806	0.413	0.025	0.463	0.300
08	10	0.271	0.515	0.387	0.391	0.094	**0.097**	0.030	0.073
	20	**0.260**	0.424	**0.340**	0.341	0.073	0.110	0.020	0.067
	50	0.281	0.355	0.370	0.335	0.069	0.114	0.016	0.066
	100	0.271	**0.287**	0.409	**0.322**	0.065	0.117	**0.013**	**0.065**
	Initial Extrinsic Parameters	0.167	0.536	6.359	2.354	0.371	0.106	0.461	0.312
12	10	1.058	**0.805**	1.307	1.056	0.109	**0.094**	0.044	**0.082**
	20	1.059	0.868	1.262	1.063	0.099	0.113	0.045	0.085
	50	1.038	0.890	1.226	1.051	0.095	0.123	0.042	0.086
	100	**1.009**	0.894	**1.193**	**1.032**	**0.093**	0.129	**0.040**	0.087
	Initial Extrinsic Parameters	0.167	0.536	6.359	2.354	0.371	0.106	0.461	0.312
14	10	0.226	**0.234**	1.071	0.510	0.160	**0.139**	0.036	0.111
	20	0.264	0.274	1.012	0.516	**0.154**	0.153	**0.025**	**0.110**
	50	0.204	0.274	0.940	0.472	0.155	0.161	**0.025**	0.113
	100	**0.171**	0.263	**0.914**	**0.449**	0.161	0.168	**0.025**	0.118
	Initial Extrinsic Parameters	0.809	2.449	2.160	1.806	0.413	0.025	0.463	0.300

**Table 7:** The calibration results given initial extrinsic parameters using the KITTI360 dataset.

Sequence	Test Length	Translation (cm)				Rotation (${}^{\circ }$)			
		$E_{X}$	$E_{Y}$	$E_{Z}$	$\bar{t}$	$E_{\mathrm{Roll}}$	$E_{\mathrm{Pitch}}$	$E_{\mathrm{Yaw}}$	$\bar{R}$
02	10	0.840	**0.211**	**1.306**	0.785	0.153	0.085	**0.037**	0.091
	20	0.787	**0.211**	1.345	0.781	0.148	0.071	0.040	0.086
	50	0.727	0.235	1.334	0.765	0.145	0.064	0.040	0.083
	100	**0.681**	0.254	1.342	**0.759**	**0.141**	**0.059**	0.039	**0.079**
06	10	0.634	**0.184**	1.049	0.622	0.214	0.068	0.033	0.105
	20	0.433	0.197	**0.996**	0.542	0.216	0.046	0.026	0.096
	50	0.359	0.215	**0.996**	0.523	0.214	**0.037**	0.021	**0.090**
	100	**0.321**	0.225	1.011	**0.519**	**0.211**	0.041	**0.018**	**0.090**
07	10	0.879	**0.339**	1.150	0.872	0.207	0.120	**0.033**	0.120
	20	0.838	0.376	**1.282**	0.861	0.184	0.116	0.038	0.112
	50	0.785	0.401	1.399	0.832	0.176	0.115	0.040	0.110
	100	**0.742**	0.409	1.465	**0.789**	**0.172**	**0.111**	0.039	**0.107**
Initial Extrinsic Parameters		3.873	0.393	1.579	1.948	5.070	2.469	0.667	2.735

## Data Availability

Not applitable.
